# Development and Validation of a New Set of Primers for Identification of Circulating Lineages and Palivizumab/Nirsevimab Resistance in HRSV Isolates from Cabo Verde

**DOI:** 10.3390/tropicalmed10060160

**Published:** 2025-06-10

**Authors:** María Paula Reyes-Zuluaga, José Antonio Pérez-Pérez, Wilson Correia, Isabel Inês M. de Pina Araújo, Emma Carmelo

**Affiliations:** 1 Instituto Universitario de Enfermedades Tropicales y Salud Pública de Canarias, Universidad de La Laguna, 38200 La Laguna, Tenerife, Spain; 2 Departamento de Bioquímica, Microbiología, Biología Celular y Genética, Facultad de Ciencias, Universidad de La Laguna, 38200 La Laguna, Tenerife, Spain; 3 Faculdade de Ciências e Tecnologia, Universidade de Cabo Verde, Praia CP 7943-010, Cape Verde; iaraujo@unicv.cv; 4 oNe hEalth Research cenTer de Cabo Verde—NEST-CV, Universidade de Cabo Verde, Praia CP 7943-010, Cape Verde; 5 Global Health and Tropical Medicine, GHTM, Associate Laboratory in Translation and Innovation Towards Global Health, LA-REAL, Instituto de Higiene e Medicina Tropical, IHMT, Universidade NOVA de Lisboa, UNL, 1349-008 Lisboa, Portugal; 6 Departamento de Obstetricia y Ginecología, Pediatría, Medicina Preventiva y Salud Pública, Toxicología, Medicina Legal y Forense y Parasitología, Universidad de La Laguna, 38200 La Laguna, Tenerife, Spain

**Keywords:** HRSV, Cabo Verde, RT-PCR, lineages, resistance, Nirsevimab, Palivizumab

## Abstract

In Cabo Verde, Acute Respiratory Infection caused by various pathogens was the most reported condition in children under 5 years old between 2014–2020, and the fourth leading cause of mortality in this age group, with Human Respiratory Syncytial Virus (HRSV) being one of the main etiological agents. However, limited literature on the subject hinders the study of its epidemiology and the evaluation of potential implications for public health. In this work, we developed and validated a primer collection for the amplification and sequencing of the G and F genes of HRSV, using a sequential workflow including conventional and semi-nested PCR, followed by Sanger sequencing. This strategy not only allowed for the identification of HRSV linages but also facilitated the detection of mutants in the HRSV F protein, a critical step towards evaluating and ensuring the continued efficacy of Nirsevimab or Palivizumab as prophylactic therapies. Our analysis revealed the presence of the HRSV lineages A.D.2.2.1, A.D.3, B.D.4.1.1, and B.D.E.1, corresponding to the globally circulating lineages during the study period (years 2019 and 2022). No previously described mutations in the F protein that confer resistance to Palivizumab and Nirsevimab were found. However, continuous monitoring of HRSV genotypes is crucial to promptly identifying resistant viruses, considering their potential impact on public health.

## 1. Introduction

Human Respiratory Syncytial Virus (HRSV) is one of the most common viral respiratory pathogens worldwide, affecting infants, young children, older adults, and immunocompromised individuals [1,2]. It is a leading cause of hospitalization in children under 2 years old due to bronchiolitis and pneumonia, causing approximately 60,000 hospital deaths globally each year in children under 5 years old [3].

HRSV is a single-stranded negative-sense RNA virus that belongs to the Pneumoviridae family and genus Orthopneumovirus [4]. Its genome is approximately 15.2 Kb in length and is organized into 10 genes that encode 11 viral proteins, consisting of 2 non-structural and 9 structural proteins. The molecular epidemiology of HRSV is quite complex due to its rapid genetic changes, leading to the emergence of numerous genotypes grouped into two main antigenic groups: subgroups A (HRSV-A) and B (HRSV-B), which circulate in different seasons and geographical regions [5,6]. The sequence of the G gene or its second hypervariable region has been used in numerous epidemiological and evolutionary studies [1,3]. However, some authors highlight that analyzing this region may not always be the best approach, since in some cases it might not distinguish whether two isolates belong to the same lineage or not [1]. Unlike the G protein, the sequences of the F ectodomains differ only by ~5% between HRSV-A and HRSV-B, rendering this an interesting target region for the development of prophylactic tools against HRSV such as the monoclonal antibodies Palivizumab and Nirsevimab [2,3]. Considerable effort has been made to classify HRSV genotypes below the subgroup level [1,7,8,9,10,11]. However, some of the traditional amplification strategies may have shown some challenges due to different amplification efficiencies for one subgroup over another due to sequence variability and dissimilar melting temperatures (Tm) of amplification primers, which can compromise both specificity and yield [12,13,14,15]. Very recently, the HRSV Genotyping Consensus Consortium (RGCC) proposed a new standardized system for the phylogenetic classification of the different HRSV genotypes, applicable to both complete and partial genomes [16]. This classification is important not only for taxonomic purposes but also to better understand the epidemiology and therefore the implementation of therapeutic and preventive strategies for this virus [1].

In recent years, there has been a growing interest in the molecular epidemiology and phylogenetic characterization of HRSV, particularly in low- and middle- income countries (LIMC), to monitor global viral evolution and identify prevailing genotypes [17,18,19,20]. However, such efforts are often hindered by the high mutation rate of HRSV and by older or poorly preserved clinical specimens. In these cases, whole-genome sequencing may be impractical due to the degradation of nucleic acids. In this context, a modular amplification strategy employing a flexible combination of PCR and sequencing primers that target critical genomic regions, while still covering the highly divergent HRSV sequences in the databases, can serve as a viable alternative. This approach enables the generation of amplicons of varying lengths, increasing the likelihood of successful amplification and sequencing from degraded or low-quality samples.

In Cabo Verde, Acute Respiratory Infection (ARI) caused by various viral pathogens, including HRSV, was the most commonly reported clinical condition in children under 5 years of age between 2014 and 2020 and the fourth leading cause of mortality in this age group [21,22]. Two recent studies by Correia et al. [23,24] indicated that HRSV is one of the primary etiological agents causing ARI in children under 5 years old in Cabo Verde.

The aim of this study was to design and validate a primer set for the amplification and sequencing of the G and F genes of HRSV. These primers were evaluated using clinical samples obtained from pediatric patients (<5 years old) presenting with acute respiratory infections and seeking hospital care in Cabo Verde during 2019 and 2022, in which HRSV had been previously detected. A sequential workflow involving conventional PCR, semi-nested PCR (as required), and Sanger sequencing of the G and F gene segments was employed. This strategy facilitated the genotypic characterization of circulating HRSV strains in Cabo Verde. This approach also enabled the detection of monoclonal antibody-resistant mutants (MARM) in the population, an important step towards monitoring the effectiveness of prophylactic therapies targeting the HRSV F protein [25].

## 2. Materials and Methods

### 2.1. Patient Samples

The clinical samples used in the present work were obtained from a study conducted with pediatric patients from Cabo Verde in 2019 and 2022 [23,24], in which the presence of HRSV virus was detected, and the viral subgroup was identified, using real-time PCR with TaqMan probes coupled with a prior reverse transcription (real-time RT-PCR) [26]. The samples were collected in the months of January, May, and November of 2019 and 2022 from children under 5 years of age who presented at the pediatric emergency department at University Hospital Dr. Agostinho Neto (UHAN) in Praia, Santiago Island, Cabo Verde, with suspected acute respiratory infection (ARI) identified by clinicians. Children were included in this study only after their guardians or parents were provided with a detailed explanation of the study’s purpose and informed consent was obtained. The exclusion/inclusion criteria for this study were described in Fitzner et al. [27] and our previous manuscripts [23,24]. Briefly, only children under 5 years of age, of both sexes, who exhibited at least three typical symptoms of ARI (nasal obstruction, cough, headache, chest pain, difficulty breathing, conjunctivitis, and/or fever ≥38 °C) with symptom onset 3–7 days before seeking medical attention and without any treatment for ARI were invited to this study. Children with severe illnesses and those in critical or terminal conditions were excluded from this study. The study was approved by the National Ethics Committee for Health Research of Cabo Verde (CNEPS, Resolution 72/2018).

The specimens consisted of nasopharyngeal swabs (NPS) collected in Viral Transport Medium (VTM) (Delta Lab, Barcelona, Spain) and stored at −80 °C until their transport to Spain. Transport was performed at 4 °C, and upon arrival to the Instituto Universitario de Enfermedades Tropicales y Salud Pública de Canarias, samples were returned to −80 °C. Seven NPS containing the HRSV-A subgroup, all from 2019, and another ten samples of the HRSV-B subgroup, nine of them collected during the 2022 sampling period, were analyzed.

### 2.2. RNA Purification and Reverse-Transcription (RT)

Viral RNA was purified using the QIAamp Viral RNA Mini Kit (Qiagen, Hilden, Germany), following the manufacturer’s instructions. RNA was recovered from the spin column in 60 µL of elution buffer. The Transcriptor First Strand cDNA Synthesis Kit (Roche, Basel, Switzerland) was used to obtain complementary DNA (cDNA) from 11 µL of total RNA samples, using random hexamers as primers. The RNA/primer mix was incubated at 65 °C for 10 min and then quickly transferred to ice. The RT reactions (20 µL) were incubated in a thermal cycler (ProFlex PCR System; Thermo Fisher Scientific, Waltham, MA, USA) with the following program: 25 °C for 10 min; 55 °C for 30 min; 85 °C for 5 min. The cDNA samples were diluted to a final volume of 40 µL using 10 mM Tris-HCl, pH 8.0.

### 2.3. Primer Design

Two reference sequences of the viral genome [8], one from the HRSV-A subgroup (Id. NC_038235.1) and another from the HRSV-B subgroup (Id. NC_001781.1), were selected as starting points for primer design. These sequences were downloaded from GenBank (NCBI) and aligned using the MEGA v11 software [28]. Highly conserved regions in the viral genome, specifically in the G and F genes, were identified through visual inspection of the alignment. The sequences of these conserved regions, including some variable positions, were evaluated as potential primer binding sites using the Primer BLAST (https://www.ncbi.nlm.nih.gov/tools/primer-blast/ (accessed on 4 June 2025)) tool from NCBI against the collection of complete HRSV genomes. This analysis allowed for the identification of additional variable positions among the sequences available in the GenBank.

The most common nucleotide variants in the conserved regions were considered for designing the set of primers (Table 1). This design was carried out using Gene Runner software v 6.5 (Hastings Software Inc., Hasting, NY, USA), avoiding too-stable secondary structures (ΔG < −5.3). The melting temperature (Tm) of the oligonucleotides was set to around 60 °C, calculated as the average of estimates from three different computational tools: Oligo Calc [29], OligoAnalyzer™ (https://eu.idtdna.com/pages/tools/oligoanalyzer; accessed on 25 January 2024) and Gene Runner. Lastly, the primers designed for DNA amplification were tested using Primer BLAST (NCBI) against the human genome reference sequence and the collection of human RNA reference sequences to predict nonspecific amplifications.

**Figure 1:**
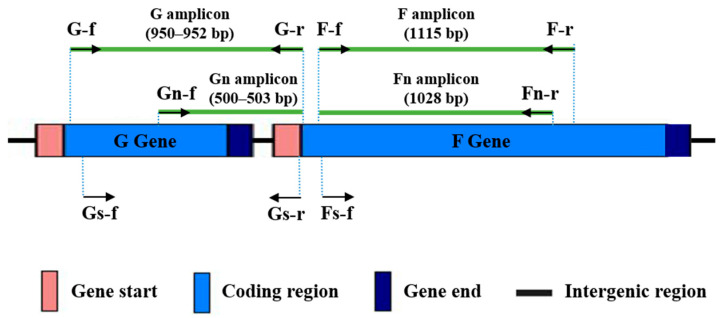
Binding sites in the HRSV genome for the designed primers. Primers are represented as arrows. Amplicon sizes in base pairs, according to the reference genomes for HRSV-A (NC_038235.1) and HRSV-B (NC_001781.1) subgroups, are shown in brackets.

The target sites for all primers in the HRSV genome are shown in Figure 1. The G amplicon is delimited by the G-f/G-r primer pair, and it spans 95.4% of the G gene coding region, from nucleotide 42 to the stop codon. The F amplicon is defined by the F-f/F-r primer pair, covering 64.6% of the F gene coding region, from positions 100 to 1214. When necessary, a semi-nested PCR was performed using the primer pairs Gn-f/G-r (Gn amplicon), targeting the second hypervariable region of the G gene, or F-f/Fn-r (Fn amplicon).

### 2.4. Polymerase Chain Reaction (PCR)

cDNA amplification assays by PCR were carried out in a final reaction volume of 20 μL containing the following: 5 μL of DNA sample; 1× reaction buffer (Thermo Fisher Scientific, Waltham, MA, USA); the 4 dNTPs at 0.2 mM each; forward and reverse primers at different concentrations depending on the fragment to be amplified (Table 1); 0.4 μL of Phire Hot Start II DNA Polymerase (Thermo Fisher Scientific, Waltham, MA, USA) and molecular biology-grade water. In the case of semi-nested PCR (Gn and Fn amplicons), a 1/10 dilution of the first PCR product after 35 amplification cycles was used as a DNA template.

The thermal profile for DNA amplification was as follows: initial denaturation at 98 °C for 30 s; 35/40 amplification cycles (15 for semi-nested PCR) with denaturation at 98 °C for 10 s, primer annealing at 55 °C for 10 s, and extension at 72 °C for 20 s (10 s with Gn amplicon); final extension at 72 °C for 20 s.

### 2.5. DNA Electrophoresis

The specificity and yield of the PCRs were verified by electrophoresis in 2% agarose gels with 1× TBE buffer, applying a constant voltage of 75V for 1 h. The molecular weight marker used was PeqGOLD DNA Ladder Mix (VWR, Radnor, PA, USA). The gels were stained with a GelRed^®^ 3× solution (Biotium, Fremont, CA, USA) for 30 min and photographed with the ChemiDoc XRS+™ imaging system (BioRad, Hercules, CA, USA).

### 2.6. Amplicon Sequencing

Excess of primers and dNTPs after PCR were removed by enzymatic treatment (ExoCleanUp FAST; VWR, Radnor, PA, USA). Cleaned amplicons were sent to an external service (Macrogen, Madrid, Spain) for automated Sanger sequencing, along with the corresponding internal sequencing primer (Table 1).

### 2.7. DNA Sequence Analysis

Sequencing electropherograms were visualized with MEGA v11 software [28]. This program was also used for nucleotide and amino acid sequences analysis, including phylogenetics inferences. These analyses were carried out separately for each HRSV subgroup.

An initial alignment of our nucleotide sequences from Cabo Verde with reference sequences for HRSV-A (NC_038235.1 and PP109421.1) and HRSV-B (NC_001781.1 and OP975389.1) was performed and subsequently translated into amino acid sequences with the standard genetic code. This allowed the identification of the amino acid signatures in the G and F protein that distinguish different lineages according to the RGCC proposal [16]. Additionally, we examined the sequence of the F protein, looking for mutations in the antibody binding sites that could confer antibody resistance.

Phylogenetic analysis of HVRS gene G was performed using one representative sequence from each lineage proposed by RGCC and the sequences obtained from the Cabo Verde samples [16]. After alignment, the ends were trimmed and maximum likelihood phylogenetic trees with 1000 standard bootstrap replicates were constructed. The selection of the most suitable nucleotide substitution models was performed with MEGA11 software according to the Bayesian Information Criterion (BIC). The TN93 + G was the most suitable model for HRSV-A, and TN93 + I for HRSV-B. Phylogenetic trees were visualized with Figtree v1.4.4 [30].

## 3. Results

### 3.1. Performance of the Designed Primers

**Figure 2:**
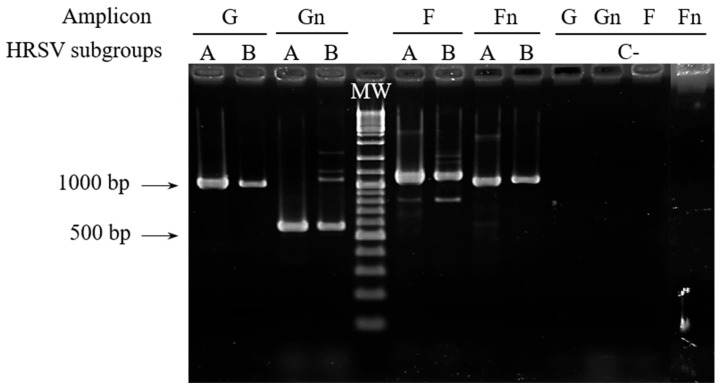
RT-PCR assays for G, Gn, F, and Fn amplicons with samples representing the two HRSV subgroups, analyzed by agarose gel electrophoresis. Results shown for G and F amplicons were obtained with 35 amplification cycles, using sample 110 for HRSV-A and sample 30 for HRSV-B as example. The arrows indicate the size in base pairs of two bands of the molecular weight (MW) marker. C−: negative controls.

The primers were used sequentially on all samples until amplicons of adequate quality and quantity were obtained for sequencing (following the flowchart included as Supplementary Figure S1). Initially, we tested G-f/G-r and F-f/F-r primer pairs with the 17 clinical samples, applying 35 cycles of PCR. In these conditions, good amplification signals (Figure 2) were obtained for G and F amplicons with 33% and 50% of the samples, respectively. Next, a second test with 40 amplification cycles was performed with those samples for which no amplification or low amplification yields were obtained in the previous PCR assays. Together, PCRs with 35 or 40 cycles provided enough quantity of amplification products with 83% of the samples for both amplicons. The remaining samples could be incorporated into the molecular analysis as Gn and Fn amplicons after performing semi-nested PCR (Figure 2). It is noteworthy that G and Gn amplicons showed a slightly larger size than predicted (around 50 bp, as observed in the agarose gel), based on the reference sequences for HRSV-A and HRSV-B (Figure 1).

Despite non-specific amplification products being observed with some samples (Figure 2), the use of internal primers allowed us to obtain good-quality sequencing electropherograms for all of them.

### 3.2. Analysis of HRSV Sequences Detected in Cabo Verde

Sequence analysis revealed that the G gene in HRSV samples from Cabo Verde is affected by a duplication of 72 nt (HRSV-A) or 60 nt (HRSV-B), which explains the size differences of the G and Gn amplicons compared to the reference genomes (Figure 1 and Figure 2). These duplications were first detected in Ontario (Canada, 2010; genotype ON1 of HRSV-A) [31] and Buenos Aires (Argentina, 1999; genotype BA of HRSV-B) [32] and have since become predominant in successive HRSV epidemics. All samples analyzed from 2019 correspond to HRSV-A [23] and those from 2022 mostly correspond to HRSV-B [24].

**Figure 3:**
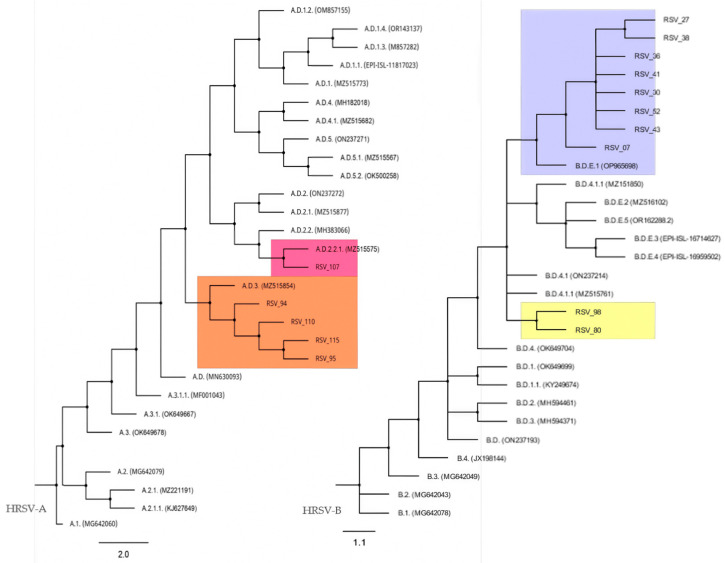
HRSV-A and HRSV-B maximum-likelihood phylogenetic tree. Color boxes highlight the positions of HRSV sequences from Cabo Verde and the nearest lineage.

The phylogenetic analysis allowed us to assign most of the nucleotide sequences from Cabo Verde to a HRSV lineage proposed by the RGCC. Four of our HRSV-A sequences clustered with lineage A.D.3, while one clustered with lineage A.D.2.2.1 (Figure 3, left). Two nucleotide sequences from HRSV-A (samples 79 and 81) were not included in the alignment because the sequence electropherograms had short unreadable regions, probably due to heterogeneity in the corresponding amplicons. On the other hand, for HRSV-B, eight sequences clustered with lineage B.D.E.1 (Figure 3, right). The HRSV-B tree presented polytomies that made it difficult to infer the phylogenetic affinity of the sequences obtained from samples 80 and 98 with a specific HRSV-B lineage, the closest being B.D.4.1. or B.D.4.1.1. The polytomies do not allow us to distinguish the branching order of certain linages, suggesting that analysis of complete genomes is required to obtain a good resolution of the phylogeny (Figure 3).

In order to more accurately identify the lineages of the HRSV sequences from Cabo Verde, we investigated amino acid signatures according to the HRSV genotype classification system proposed by the RGCC [16]. Attending to these signatures, samples of the HRSV-A subgroup from Cabo Verde mostly correspond to the lineage A.D.3 (six genotypes) and only one of them to the lineage A.D.2.2.1, whereas six samples of HRSV-B subgroup belong to the lineage B.D.E.1, as previously revealed by the phylogenetic analysis. However, with this new analysis, amino acid signatures detected in HRSV-B sequences from samples 98 and 80 allowed us to assign them to the lineage B.D.4.1.1 (Table 2 and Supplementary Table S1).

To suggest a possible origin of the genotypes found in Cabo Verde, the nucleotide sequences we obtained were compared with those available in GenBank and corresponding to HRSV samples collected worldwide in the years 2019 and 2022. In this sense, the highest levels of sequence identity were up to 99.74% for the G gene and 100% for the F gene with respect to HRSV genotypes mainly from the USA, Germany, and China (Supplementary Table S1).

### 3.3. Amino Acid Changes in the F Protein

Monoclonal antibodies targeted to one of the six antigenic sites (Ø, I–V) of the F protein have been successfully used as a passive immunoprophylactic strategy to prevent severe HRSV infections in infants [33,34,35]. The analysis of amino acid substitutions in the fusion protein revealed that mutations Asn262Ser/Tyr/Asp, Lys272Glu/Asn/Met/Gln/Thr, and Ser275Phe/Leu, which confer resistance to the neutralization by Palivizumab [36,37,38,39], were not found in the sequences obtained from Cabo Verde samples. However, the mutation Asn276Ser adjacent to the Palivizumab binding site was found in all our HRSV-A sequences.

Regarding Nirsevimab binding sites, none of the mutations on site II that confer reduced susceptibility to this antibody (Asn67Ile + Asn208Tyr for HRSV-A and Asn208Ser/Asp, Lys68Asn + Asn201Ser, Lys68Asn + Asn208Ser, Ile64Met + Lys65Glu or Ile64Thr + Lys68Glu + Ile206Met + Gln209Arg for HRSV-B) were found in the analyzed sequences [40,41]. However, the mutations Ile206Met and Gln209Arg in the Ø epitope were detected in all HRSV-B sequences analyzed, and the substitution Ser211Asn in the same epitope was observed in 70% of them.

## 4. Discussion

HRSV has been a longstanding focus for the WHO, with efforts aimed at strengthening surveillance—especially among infants and young children—expanding virologic monitoring to differentiate virus types and identify genetic groups, and improving the understanding of its epidemiology, particularly in low- and middle-income countries across all WHO regions [42]. Therefore, monitoring HRSV molecular epidemiology in a low-income country like Cabo Verde is crucial for guiding effective public health responses, optimizing resource allocation, and informing vaccine strategies tailored to local viral strains and transmission dynamics.

In recent years, several partners in Cabo Verde and Spain, including health professionals from University Hospital Dr. Agostinho Neto (the main hospital run by the Ministry of Health of Cabo Verde), have conducted studies to determine the epidemiological and clinical profile of viral respiratory infections in children under 5 years old [23,24]. However, these efforts are sometimes hampered by poor preservation of clinical samples, particularly in low-resource settings, an issue that demands a modular, sequential approach to overcome nucleic acids degradation or low viral loads. In this context, the aim of this study was to design and validate a collection of primers for the amplification and sequencing of the G and F genes of HRSV that allow the identification of HRSV isolates below the subgroup level, as well as the detection of MARM in the HRSV F protein. Our results indicate that adjusting PCR conditions, such as sequentially increasing the number of amplification cycles and performing semi-nested PCR, was key to improving the reaction’s performance and to obtaining satisfactory outcomes with the different samples. By adjusting the method to account for sample quality and/or viral load, high-quality sequence reads were obtained, enabling lineage determination and providing sufficient data for monitoring the emergence of MARMs.

Given that the nasopharyngeal samples used in this study were collected in Cabo Verde in 2019 and 2022, molecular analysis faced another challenge due to the high mutation rate of this RNA virus, which may render some PCR strategies unsuitable for genotyping more recent isolates. This new primer set was specifically designed to span both older reference genotypes and more recent ones, incorporating degenerate bases and intentional mismatches to enable amplification of both subgroups with similar efficiency, paying attention to sequence variability among those available in GenBank. Although the designed primers worked relatively well with old samples—from 2019 and 2022—experience indicates that the genetic material is best preserved as cDNA, so it is recommended to purify viral RNA and perform reverse transcription reactions as soon as possible after sample reception.

Even though several authors recommend the use of complete HRSV genomes for better lineage assignment and monitoring of amino acid changes, other authors indicate that partial genomes containing the G and F genes can be used because they exhibit a robust phylogenetic signal due to surface glycoproteins that are likely under selection pressure from antibody-mediated immunity [16]. Consistent with this perspective, the present study analyses the G and F proteins to identify the HRSV lineages circulating in Cabo Verde during 2019 and 2022 based on amino acid signature. In 2019, HRSV-A lineages were more prevalent globally than HRSV-B, including lineage A.D.2.2.1, which has not been observed in subsequent years, and lineage A.D.3—both of which were identified in our samples. In 2022, lineage B.D.E.1 was the most frequently detected globally among both HRSV subgroups, and, along with B.D.4.1.1, was also found in the Cabo Verdean samples.

The recent introduction of novel prophylactic strategies in some countries—such as monoclonal antibodies targeting specific epitopes of the HRSV F protein—underscores the importance of closely monitoring mutations in these regions that may compromise therapeutic efficacy. Although neither Palivizumab nor Nirsevimab are currently employed in Cabo Verde, the country’s socio-economic context, characterized by high migration flows from regions including West Africa, Portugal, Italy, Brazil, the United States and Asia, as well as a substantial foreign resident population of approximately 11,000 individuals, demands vigilance in order to detect the potential emergence of antibody-resistant viral variants [43,44]. Focusing on amino acid substitutions of interest in the fusion protein, the amino acid at position 276, adjacent to the Palivizumab binding site, is generally asparagine in HRSV-A and serine in HRSV-B [45,46]. The Asn276Ser mutation in the F protein of HRSV-A was first reported in 2007 and was found in all our HRSV-A sequences from Cabo Verde. Although it does not generate resistance to Palivizumab by itself [37,46], amino acid 276 is thought to be susceptible to selection pressure by the prophylactic antibody, considering that the binding capacity of Palivizumab may be completely lost when, after the Asn276Ser mutation, a second amino acid change is introduced at the site recognized by the antibody. Hence, it is considered as the first amino acid mutation inducing MARM, highlighting the need to monitor the local evolution of the HRSV F gene [37,45,46].

Regarding the mutations in the Ø epitope detected in all the HRSV-B sequences, Ile206Met mutation alone reduces the neutralization capacity of Nirsevimab 5-fold, but it usually occurs together with the Gln209Arg mutation, and this combination shows greater susceptibility to neutralization by this monoclonal antibody [47,48]. In conclusion, the combination of mutations Ile206Met + Gln209Arg in the Nirsevimab binding site found in our sequences does not affect the susceptibility of the virus to neutralization by this drug [49]. Given that HRSV is a single-stranded RNA virus with a high mutation rate, rapid genetic changes might be expected to arise upon extensive use of Nirsevimab or Palivizumab. This, again, highlights the importance of closely monitoring circulating HRSV genotypes in order to detect early the emergence of resistant viruses to this drug, given the implications that this would have for public health.

Despite the valuable information obtained in this study, there are some limitations that deserve consideration. First, the study was conducted over a three-month period over two years and only included children attending the emergency department for ARI, so the limited sample size restricts the ability to draw conclusions for the broader population. Second, our methodology does not allow for complete sequencing of the viral genome, which may result in the loss of some relevant epidemiological information.

In conclusion, this work developed and validated a primer collection for the amplification and sequencing of the G and F genes of HRSV, enabling the genotypic characterization of viral strains circulating in Cabo Verde. Using clinical samples from pediatric patients under five years of age with acute respiratory infections collected in 2019 and 2022, the study employed a sequential workflow including conventional and semi-nested PCR, followed by Sanger sequencing. This strategy not only allowed for the identification of HRSV linages but also facilitated the detection of mutations in the HRSV F protein, critical information for evaluating and ensuring the continued efficacy of Nirsevimab or Palivizumab as prophylactic therapies.

## Figures and Tables

**Table 1:** Oligonucleotides used as primers.

Name ^a^	Sequence ^b^	Purpose	Amplicon	Tm (°C) ^c^	Concentration (µM) in PCR
G-f	A**R**AAGACCTGGGATACTCT**Y**AATC	PCR	G	59.0–62.0	0.4
G-r	GGAT**Y**GGCAACTCCAT**K**GTTATTT	PCR/semi-nested PCR	G/Gn	60.8–62.0	0.4
Gn-f	CA**Y**TTTGAAGTGTTCAA**Y**TT**Y**GT**D**CC	Semi-nested PCR	Gn	61.6–64.3	2
Gs-f	C**WR**TT**Y**TGGCAATGATAATCTCAAC	Sequencing	G	57.8–61.4	―
Gs-r	GTTATTTGCCCCAGA**K**TT**R**ATTT**Y**G	Sequencing	Gn	62.0–63.4	―
F-f	CAATC**R**ACATGTAGTGCAGTTAGC	PCR/semi-nested PCR	F/Fn	60.0–61.4	0.2
F-r	GAGCTGCTTA**YR**TCTGTTTTTGA	PCR	F	60.2	0.4
Fn-r	GGTAATGT**Y**AAACTGTTCAT**W**GTGTCAC	Semi-nested PCR	Fn	63.3–63.9	0.6
Fs-f	GCAGTTAGCA**R**AGG**Y**TAT**Y**T**K**AGTG	Sequencing	F/Fn	60.7–62.8	―

^a^ f: forward; r: reverse. The F-r primer is a slightly modified version of another one taken from the literature [15]. ^b^ Degenerated positions are highlighted in bold: D = A, T o G; K = G o T; R = A o G; W = A o T; Y = C o T. Underlined positions involve mismatches with the target sequence in one of the HRSV subgroups. ^c^ Melting temperature range considering the vast majority of available HRSV genome sequences.

**Table 2:** HRSV lineages detected in patients from Cabo Verde according to the standards established by the RGCC [16].

Subgroup	Lineage	Identified Amino Acid Signatures ^a^	
		G Protein	F Protein
HRSV-A	A.D.2.2.1	Glu224Gly	n.a.
	A.D.3	Thr113Ile; Val131Asp; Asn178Gly; His258Gln; His266Leu	n.a.
HRSV-B	B.D.4.1.1	Thr288Ile	Lys191Arg; Ile206Met; Gln209Arg
	B.D.E.1	Ser100Gly; Pro214Ser; Pro211Leu; Ile252Thr; Lys256ASn; Ile268Thr; Ser275Pro; His285Tyr	Ser190Asn; Ser211Asn

^a^ Positions and amino acid substitutions compared to reference genomes for HRSV-A (PP109421.1) and HRSV-B (OP975389.1). n.a.: not applicable/available.

## Data Availability

Data available on request from the authors.

## Supplementary Materials

The following supporting information can be downloaded at: https://www.mdpi.com/article/10.3390/tropicalmed10060160/s1, Table S1: Sequence identity of HRSV lineages detected in Cabo Verde compared with Global HRSV surveys in 2019 and 2022; Figure S1: Algorithm for genetic analysis of HRSV samples. This figure presents a detailed flowchart outlining the steps involved in the genetic analysis of Human Respiratory Syncytial Virus (HRSV) samples.

## Abbreviations

HRSV	Human Respiratory Syncytial Virus
ARI	Acute Respiratory Infection
RGCC	RSV Genotyping Consensus Consortium
RT-PCR	Polymerase chain reaction with reverse transcription
cDNA	Complementary DNA
NCBI	National Center for Biotechnology Information
BLAST	Basic Local Alignment Search Tool
