# Supplementary material for: Development and Validation of a New Set of Primers for Identification of Circulating Lineages and Palivizumab/Nirsevimab Resistance in HRSV Isolates from Cabo Verde
Source: Trop Med Infect Dis. 2025 Jun 10;10(6):160. doi: 10.3390/tropicalmed10060160 (PMC12197772; doi:10.3390/tropicalmed10060160)
Supplement: Supplementary file 1 [file tropicalmed-10-00160-s001.zip › tropicalmed-3615330-supplementary.pdf]

Supplementary Materials

Figure S1. Algorithm for genetic analysis of HRSV samples. This figure presents a detailed flowchart out-lining the steps involved in the genetic analysis of Human Respiratory Syncytial Virus (HRSV) samples.

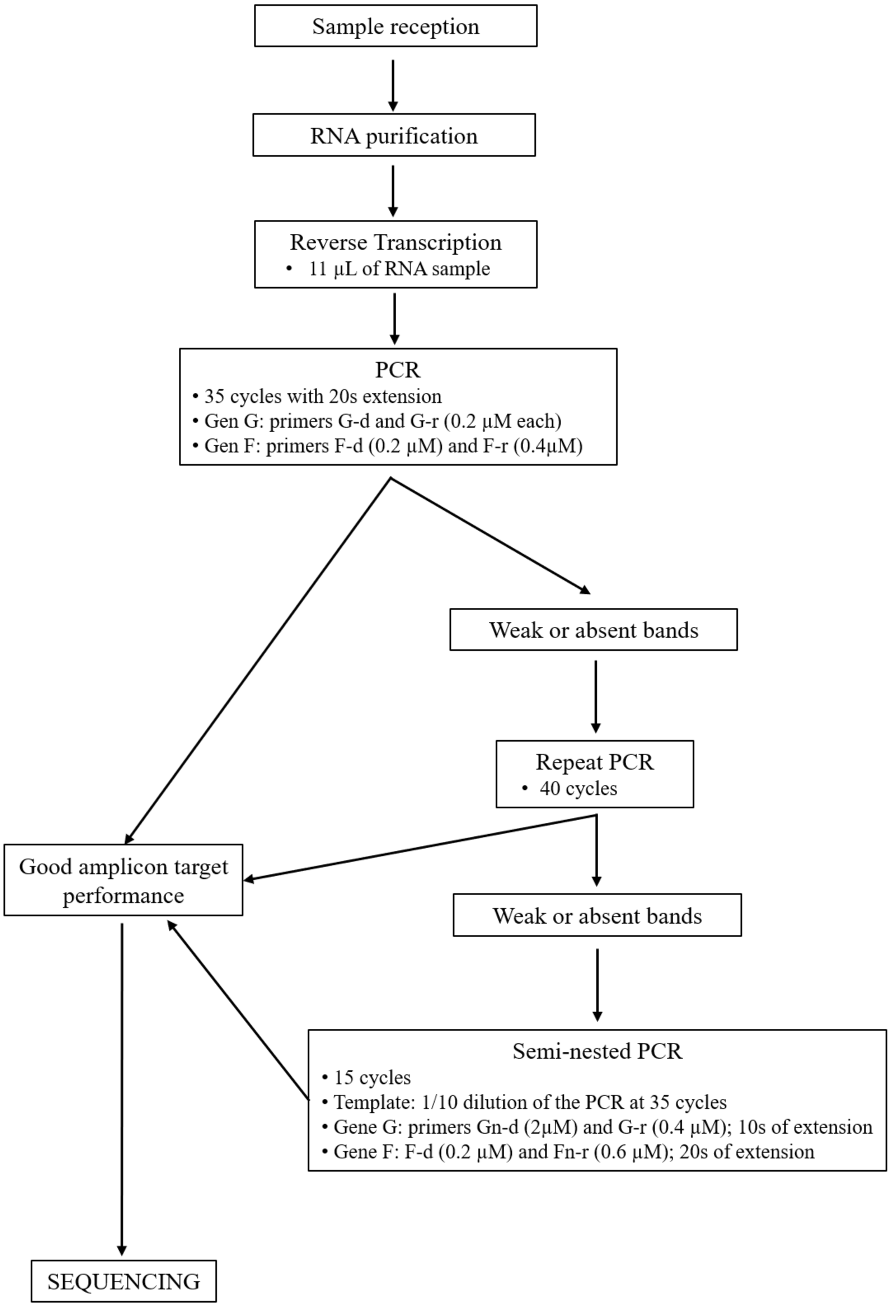

Table S1. Sequence identity of HRSV lineages detected in Cabo Verde compared with Global HRSV surveys in 2019 and 2022.

Subgroup	Lineage	Sample	Closest identity	
			**F gen**	**G gen**
**HRSV-A**	**A.D.2.2.1**	**107**	99.89%: PP376358.1 (Germany) ; MZ515852.1 (Netherlands) ;	99.50%: PP376639.1 (Germany) ; PP376431.1 (Germany)
	**A.D.3**	**79**	99.87%: ON057067.1 (USA) ; OQ277297.1 (USA)	*98.69%: OR666539.1 (China)
		**81**	99.22%: ON057067.1 (USA) ; OQ277297.1 (USA)	*99.09%: OR666539.1 (China)
		**94**	99.63%: ON057067.1 (USA) ; OQ277297.1 (USA)	99.37%: OR666539.1 (China)
		**95**<br>**110**<br>**115**	100%: ON057067.1 (USA) ; OQ277297.1 (USA)	99.50%: OR666539.1 (China)
**HRSV-B**	**B.D.E.1**	**07**	99.89%: PQ349069.1 (United Kingdom); OR975315.1 (USA)	99.51%: OR795285.1 (Germany) ; PQ348915.1 (United Kingdom)
		**27**	99.52%: PQ638733.1 (USA) ; PP760417.1 (USA)	98.47%: OR795291.1 (Germany) ; OR143243.1 (USA)
		**30**<br>**38**<br>**52**	100%: PP760418.1 (USA) ; PP760417.1 (USA)	99.74%: OR143243.1 (USA)
		**36**	99.81%: PP084847.1 (Germany) ; PQ638733.1 (USA)	99.36%: OR143243.1 (USA)
		**41**	97.85%: PP760417.1 (USA) ; PQ638734.1 (USA)	99.36%: OR143243.1 (USA) ; PP495978.1 (USA)
		**43**	99.67%: PQ638733.1 (USA) ; PP760417.1 (USA)	99.10%: OR143243.1 (USA) ; PP495978.1 (USA)
	**B.D.4.1.1**	**80**	99.69%: OQ278311.1 (Australia) ; PP377183.1 (Australia)	99.36%: MZ516113.1 (Netherlands) ; PP376993.1 (Netherlands)
		**98**	99.90%: OQ278479.1 (Netherlands) ; OP320404.1 (Philippines) ; OQ278592.1 (France) ; OQ280008.1 (USA)	98.21%: MZ516113.1 (Netherlands) ; PP376993.1 (Netherlands)

GenBank accession numbers and closest percentage identities to our sequence are indicated. Information obtained only with data from 2019 and 2022.

* Data obtained with second hypervariable region of gen G.
